# Genomic analysis of the early COVID-19 pandemic in Haiti reveals Caribbean-specific variant dynamics

**DOI:** 10.1371/journal.pgph.0003536

**Published:** 2024-11-20

**Authors:** Alexandra Mushegian, Allie Kreitman, Martha I. Nelson, Matthew Chung, Christopher Mederos, Allison Roder, Stephanie Banakis, Anne Marie Desormeaux, Nadia Lapierre Jean Charles, Yoran Grant-Greene, Samson Marseille, Katilla Pierre, Donald Lafontant, Jacques Boncy, Ito Journel, Josiane Buteau, Stanley Juin, Elodie Ghedin

**Affiliations:** 1 Systems Genomics Section, Laboratory of Parasitic Diseases, Division of Intramural Research, National Institute of Allergy and Infectious Diseases, National Institutes of Health, Bethesda, Maryland, United States of America; 2 Computational Biology Branch, National Center for Biotechnology Information, National Library of Medicine, NIH, Bethesda, Maryland, United States of America; 3 CDC Country Office, Port-au-Prince, Haiti; 4 Direction d’Epidémiologie de Laboratoire et de Recherche, Port-au-Prince, Haiti; 5 Laboratoire National de Santé Publique, Port-au-Prince, Haiti; University of Alberta, CANADA

## Abstract

Pathogen sequencing during the COVID-19 pandemic has generated more whole genome sequencing data than for any other epidemic, allowing epidemiologists to monitor the transmission and evolution of SARS-CoV-2. However, large parts of the world are heavily underrepresented in sequencing efforts, including the Caribbean islands. We performed genome sequencing of SARS-CoV-2 from upper respiratory tract samples collected in Haiti during the spring of 2020. We used phylogenetic analysis to assess the pandemic dynamics in the Caribbean region and observed that the epidemic in Haiti was seeded by multiple introductions, primarily from the United States. We identified the emergence of a SARS-CoV-2 lineage (B.1.478) from Haiti that spread into North America, as well as evidence of the undocumented spread of SARS-CoV-2 within the Caribbean. We demonstrate that the genomic analysis of a relatively modest number of samples from a severely under-sampled region can provide new insight on a previously unobserved spread of a specific lineage, demonstrating the importance of geographically widespread genomic epidemiology.

## Introduction

During the COVID-19 pandemic, genome sequencing of SARS-CoV-2 for surveillance of viral diversity was implemented on a scale never seen in prior epidemics, enabling global monitoring of virus evolution and transmission dynamics. International efforts allowed both epidemiological investigation of local outbreaks and conclusions about global patterns. For example, studies showed that outbreaks after introduction into new countries varied widely in size [1] and revealed a cryptic circulation of the virus in the winter of 2019–2020 [2]. Importantly, these efforts also highlighted the strengths and weaknesses in the global scientific community’s ability to develop the technical and analytical capacity to generate and share these data. The lessons learned can be applied to developing genome sequencing infrastructure for future pandemics.

One major conclusion from overviews of global SARS-CoV-2 genome sequencing efforts is that sequence data generation is unevenly distributed globally [3]. A region with a particularly stark underrepresentation of genome sequences in global repositories is Central America and the Caribbean islands. The Caribbean is a populous region (>40 million people in the island states) at the crossroads of trade routes and visited by tourists from around the world, with highly heterogeneous socioeconomic conditions and healthcare systems. The relative isolation of islands can result in highly idiosyncratic infection dynamics compared to contiguous locations at similar distances.

We performed whole genome sequencing of SARS-CoV-2 samples collected in the spring of 2020 in Haiti, the most populous country in the Caribbean. We used phylogenetic analysis to gain insight into the early pandemic in the country and to glimpse into the dynamics in the broader region. In Haiti, the first cases of COVID-19 were identified in March 2020, approximately at the same time as the borders of Haiti and its adjoining Dominican Republic were closed, and many Haitians working in the Dominican Republic tourism industry returned home; cases rose rapidly in May 2020. Although accurate estimates of COVID-19 case counts in 2020 were hampered by limited healthcare capacity, natural disasters, and political unrest, the public health burden of the pandemic was considered to be less than initially feared [4]. We sought to investigate whether the epidemic in Haiti during those first months was seeded by a single or multiple introductions into the country and the extent of epidemiological linkages with other global regions that could be inferred from the genomic data.

## Results and discussion

We aimed to increase the number of SARS-CoV-2 genomic sequences publicly available for Haiti from the early months of the pandemic. The global repository GISAID (Global Initiative on Sharing All Influenza Data), as of February 2023, contained only 183 high quality genome sequences of SARS-CoV-2 collected in the region in the first six months of 2020 (Table 1). During this period, global tourism was abruptly halted, and major social and economic disruptions occurred.

**Table 1:** GISAID Entries from Caribbean islands. Summary of number of samples by Caribbean country publicly available on GISAID, toggled as complete (sequences >29,000nt), high coverage (sequences with <1% Ns and <0.05% unique amino acid mutations), and collection date compete, for first 6 months of 2020, available as of February 2023, including the samples reported in this paper, which were collected in this timeframe and are not excluded by the GISAID filter of complete, high coverage, and collection date complete.

Country	Number of sequences in GISAID
Aruba	6
Cuba	2
Curacao	6
Dominican Republic	8
Guadeloupe	26
Haiti, this study	33
Haiti, other studies	36
Jamaica	8
Puerto Rico	21
Saint Barthelemy	2
Saint Martin	10
The Bahamas	4
U.S. Virgin Islands	21

**Fig 1:**
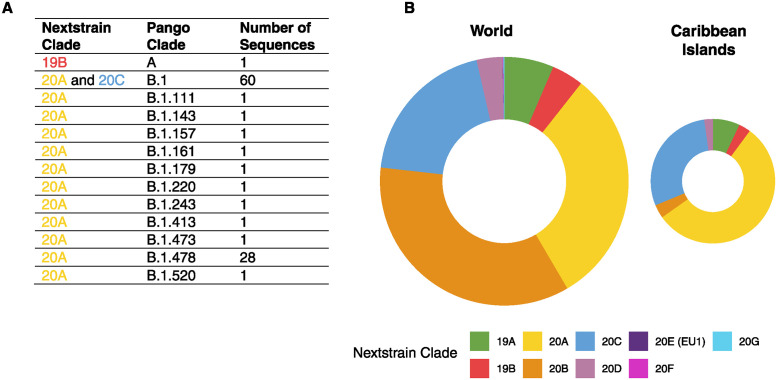
Fraction of SARS-CoV-2 sequences by nextclade lineage in global dataset versus Caribbean dataset. **(A)** Count of samples sequenced from Haiti by their Pango classification. **(B)** The fraction of SARS-CoV-2 sequences of different lineages in the global dataset of SARS-CoV-2 sequences versus a Caribbean specific dataset (S2 Table). Color represents viral lineage, called by Nextclade [7].

Of the 99 samples sequenced for this project with sufficient coverage to be classified using the Pango classification [5], the majority (60%) were classified in lineage B.1, the large European lineage that was the ancestor of all future variants of concern; sublineage classification could however not be made (Fig 1A). A significant number (28%) were classified as Pango lineage B.1.478, a rare lineage that was previously found primarily in the United States. Interestingly, one of the earliest samples (March 21, 2020) was classified in the Pango A lineage, one of the original haplotypes of the virus that made up ~30% of global infections in January 2020 and that quickly dropped to a negligible fraction of cases, making up less than 2% of samples sequenced in March 2020 (queried from cov-spectrum.org [6] in July 2022). The other two March samples, from March 18, were classified as lineage B.1.157 and B.1.220, different sublineages of the B.1 clade (S1 Table). These results suggest that there were multiple introductions of SARS-CoV-2 into the country very early in the pandemic.

To further investigate the pattern of infections in the spring of 2020, we constructed a maximum likelihood phylogeny of samples with coverage over at least 25,000 nt (75 samples) on a background of all available samples from the Caribbean and randomly selected samples from all other global regions (sampling described in Methods). Because the sampling period was early in the pandemic when overall diversity was relatively low, the resulting tree contained many unresolved polytomies, but also several clear monophyletic clades corresponding to country-specific outbreaks. Samples from Haiti span a wide range of the circulating global diversity of viruses from the first half of 2020 (Fig 1B, S1 Fig; files for viewing the tree interactively in the Auspice and Taxonium visualization servers are provided as S1 and S2 Files). The earliest samples placed in the tree, from March 18 and 21, were most closely related to samples from Scotland and from Hong Kong, respectively.

**Fig 2:**
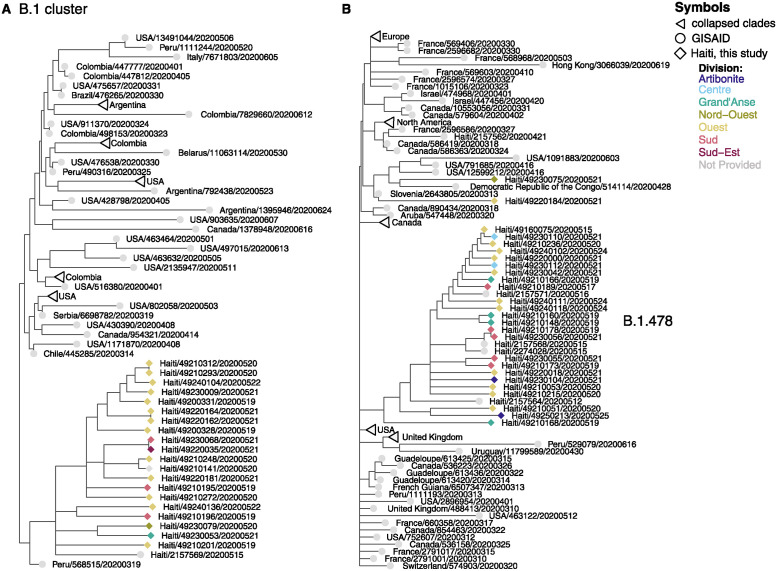
Two most highly represented SARS-CoV-2 lineages from Haiti. Maximum likelihood phylogenetic trees inferred from SARS-CoV-2 viruses from January 2020 through June 2020. Tips are shaded by Division in Haiti, and country of origin is indicated in the sample name. Triangles represent clades that were collapsed because they did not contain any Haiti data, circles represent samples downloaded from GISAID, and diamonds represent samples sequenced for this study. **(A)** Includes a cluster of Haiti samples from the pangolin B.1 lineage and **(B)** includes a cluster of Haiti samples from the B.1.478 pangolin lineage.

All samples from Haiti previously deposited in GISAID closely clustered with the sequences from this study, which also represented several new clusters of various sizes throughout the background tree. Many clusters were small (<5 sequences), but two large monophyletic clusters of Haiti samples were identified: a cluster of 21 B.1 samples and a cluster of 28 B.1.478 samples. In both clusters, samples came from at least 5 different departments in the country, suggesting that the identification of large clusters is not due to biased sampling from a single location (Fig 2). These patterns suggest that even with relatively limited sampling, it was possible to identify multiple introductions of the virus, some of which led to widespread transmission throughout the country. The B.1 cluster was found in a clade containing a mixture of North, South, and Central American samples (Fig 2A), and the B.1.478 cluster was within a clade of primarily North American and European samples (Fig 2B). In both regions of the tree, polytomies were extensive.

**Fig 3:**
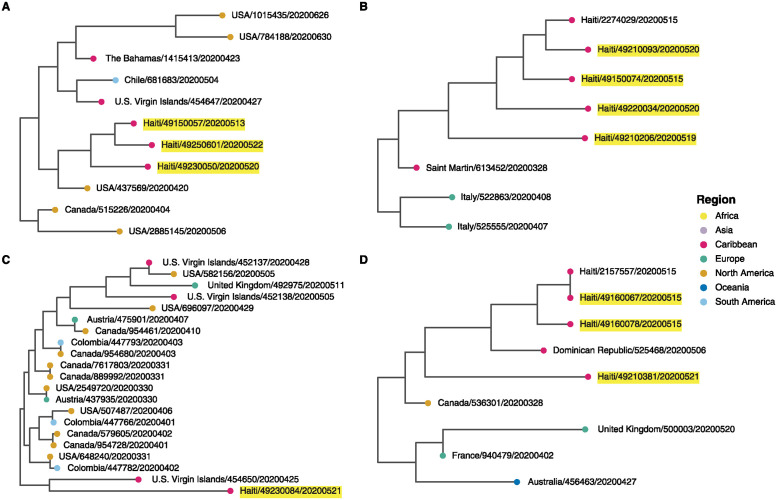
Four inter-Caribbean SARS-CoV-2 phylogenetic clusters. Maximum likelihood phylogenetic trees inferred from SARS-CoV-2 viruses from January 2020 through June 2020. Tips are shaded by region of sample collection, country of origin is indicated in the sample name, and samples sequenced for this study are highlighted in yellow. **(A-D)** represent four cases of inter-Caribbean SARS-CoV-2 clusters.

At least four locations on the tree showed that the new Haiti sequences were closely related to samples from other Caribbean locations that were previously linked to very few other Caribbean samples. For example, sequences from the U.S. Virgin Islands, Saint Martin, the Bahamas, and the Dominican Republic, whose closest relatives prior to this analysis were sequences from Chile, Italy, and the United States, now have close relatives in the Haiti samples (Fig 3). While it is not possible to ascertain whether these linkages represent direct transmission, they hint at the existence of unsampled Caribbean transmission chains.

**Fig 4:**
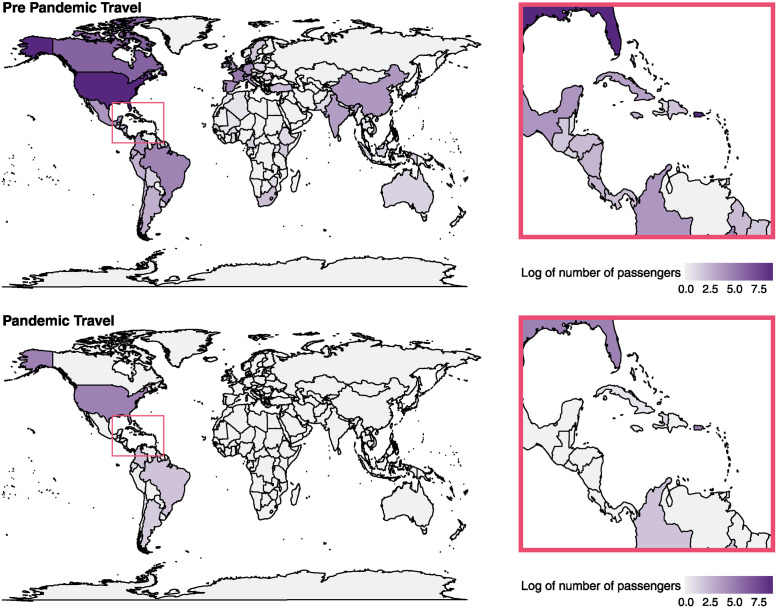
International air travel loads before and after the pandemic. Log of the average number of passengers traveling from an origin country to Haiti are plotted on a world map. Country color indicates the log of the number of passengers traveling to Haiti per month. **(A)** Plots travel patterns in January to February 2020 and **(B)** plots travel patterns in April to May 2020.

International travel has seeded the establishment of new SARS-CoV-2 variants throughout the pandemic. Thus, we analyzed passenger air travel volumes from January 2020 through July 2020 arriving in Haiti (Fig 4). In early 2020, before the pandemic, Haiti received over 30,000 travelers per month from outside the Caribbean islands and Central America. Approximately 88% of this travel originated in North America, 6% in South America, 5% in Europe, and the rest from a combination of Asia, Africa, and Oceania. After the pandemic began and travel restrictions were put into place, all travel from outside of the Americas stopped, and the remaining travel originated from the Americas, including many small Caribbean and Central American Countries. Before the pandemic, less than 5% of flights to Haiti originated within the Caribbean islands, whereas at the beginning of the pandemic, flights originating from the Caribbean made up almost 15% of flights to Haiti. While the volume of flights from the US decreased by approximately 99% at the beginning of the COVID-19 pandemic in April and May compared to just before the pandemic began, it remained the largest origin of flights to Haiti, making up 90% of total international flights into Haiti (S3 Table). The high volume of international travel into Haiti before the COVID-19 pandemic supports the potential for international importations of SARS-CoV-2 before widespread travel restrictions were implemented, including flights from the United Kingdom and China, the closest ancestors of the earliest Haiti samples we sequenced (S1 Fig). Then, inter-Caribbean spread of SARS-CoV-2 and transmission primarily within the Americas during the Spring of 2020 is supported by evidence of travel occurring almost exclusively within those regions.

**Fig 5:**
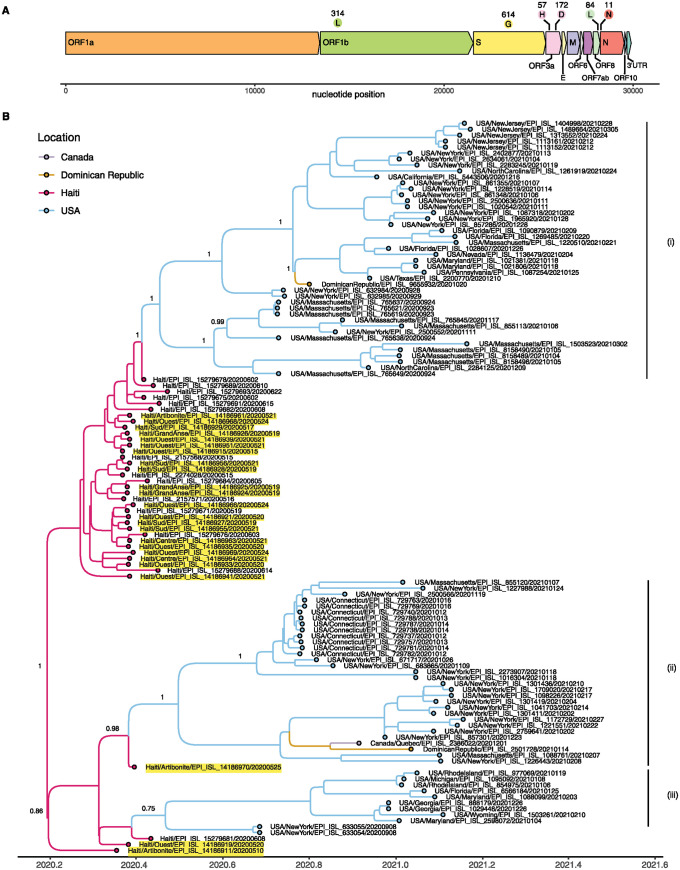
Ancestral State Reconstruction of B.1.478 lineage. **(A)** Clade defining amino acid changes in the SARS-CoV-2 B.1.478 lineage. **(B)** Bayesian timescale MCMC tree inferred for the cluster of B.1.478 SARS-CoV-2 viruses. Tips are shaded by country of origin, branches are shaded by the state inferred from an ancestral state reconstruction, and samples sequenced for this study are highlighted in yellow. Posterior probabilities are provided for key nodes.

Within Haiti, the large percentage of samples classified as B.1.478 led us to further investigate this candidate Caribbean-specific lineage. This lineage is related to the widespread B.1 lineage, and is defined by nonsynonymous mutations in *ORF1b*, *S*, *ORF3a*, *ORF8*, and *N* genes (Fig 5A). Prior to this sequencing study, 89 samples with this classification were available in GISAID spanning May 2020 to March 2021, of which one was collected in Canada, 24 in Haiti, 2 in the Dominican Republic, and 82 in the US (33 from New York, 14 from Massachusetts, 10 from Connecticut, and the remainder from 12 additional states). This lineage was, therefore, extremely rare in the United States, albeit with a wide geographic range and timeframe (May 2020-March 2021) that suggest repeated introductions into the US prior to the emergence of the Alpha (B.1.1.7) variant as the dominant lineage. In contrast, our spring 2020 sampling in Haiti revealed B.1.478 as one of the most common lineages, representing nearly 25% of the limited viral sequences available from the Caribbean in that timeframe, suggesting that it is highly overrepresented in Haiti specifically or the Caribbean generally.

To determine whether this lineage was most likely to have originated in the US or the Caribbean, we constructed a Bayesian timescale MCC tree of all available B.1.478 sequences (Fig 5B). The United States has the largest number of B.1.478 sequences (n = 82), but the earliest B.1.478 viruses were sampled in Haiti in May-June 2020, with B.1.478 not detected in the United States until September 2020. Despite the lower availability of B.1.478 samples from Haiti, the MCC tree strongly supports a B.1.478 origin in Haiti (posterior probability >0.99 for the root node). The MCC tree also indicates that B.1.478 viruses circulated undetected in Haiti for several months prior to the collection of the first B.1.478 sample on May 10, 2020, based on the inferred time of the root node (January 18, 2020). This analysis suggests that after circulating in Haiti, B.1.478 viruses were introduced into the United States on three occasions during the summer of 2020 leading to the circulation of three distinct B.1.478 clades (Fig 5B i, 5B ii and 5B iii). These three clades seemingly spread across the US Northeast region during the fall of 2020 and in some cases were identified in other US regions, Canada, and the Caribbean. Notably, the two B.1.478 viruses identified in the Dominican Republic do not appear to have been imported directly from Haiti (Fig 5B i and 5B ii), even though Haiti and the Dominican Republic share a border. Rather, the Dominican Republic B.1.478 viruses appear to have been imported from the United States on two separate occasions during the fall of 2020, representing two independent Haiti-to-US-to-Dominican Republic migration pathways. At the time of these US to Dominican Republic introductions, in the last quarter of 2020, Haiti had stricter quarantine measures in place than the Dominican Republic, which required only screening [8, 9]. While the US had a ban on travelers from high-risk regions entering the country, passengers were still flying out of the US to other countries. Just over 100x more passengers flew from the US to the Dominican Republic (317,743) than from Haiti to the Dominican Republic (3,117) between October 2020 and December 2020, which provides a potential route of transmission for the B.1.478 lineage from the US to the Dominican Republic.

A limitation of our study is that in the absence of traditional epidemiological data, we have drawn inferences based on genomic evidence. Bayesian MCMC methods are sensitive to sampling effort, so analyses of the directionality of transmission should be interpreted with caution. A number of samples had sufficient coverage to assign lineage classifications but insufficient coverage for more detailed genomic analyses, further limiting the scope of possible inference. However, even broad classification of these new sequences clearly reveals the presence of previously unknown introductions and transmission chains.

## Conclusion

In this study, we generated sequence data that more than doubled the number of whole genomes available for the first six months of 2020 in Haiti. The absolute number of samples we added was small compared to the scale of sequencing in other parts of the world, but this increase is nevertheless sufficient to illustrate previously unknown global transmission dynamics. We demonstrate that SARS-CoV-2 was introduced multiple times into Haiti, likely from both North and South America and possibly even further locations, and it was spreading between Caribbean islands in the spring of 2020. It also highlights a Caribbean-specific lineage (B.1.478) that could have initially emerged in Haiti and was introduced from the Caribbean into North America three times, where it was detectable over a period of several months. The fact that fewer than 80 samples from a severely under-sampled region could shed light on a previously unobserved regional and international spread of a specific lineage demonstrates the importance of geographically widespread genomic epidemiology.

We believe the marginal utility of additional sequencing from under-sampled regions far exceeds the benefit of additional sequencing in well-represented regions; thus, attempts should be made to expand resources and sequencing capacity to a broader range of geographic and social contexts, even if the scale of the sequencing efforts remains modest in these underserved areas. Striving for a more representative random sampling of pathogen genome sequencing, rather than opportunistically sequencing in locations where capacity already exists, should be a high priority for the future of genomic monitoring as part of pathogen surveillance efforts.

## Methods

### Sampling and sequencing criteria

At the beginning of the COVID-19 outbreak, Haiti’s surveillance system was primarily focused on collecting samples from travelers coming from abroad who displayed COVID-19 symptoms or were in contact with infected individuals. However, as the outbreak progressed, the number of suspected cases increased significantly in many communities. The Ministry of Public Health and Population (MSPP) initially relied on the laboratory-based surveillance system PRESEPI, which was already conducting surveillance for flu and flu-like diseases, to collect samples from the ten geographic departments of Haiti. However, due to the high number of symptomatic patients in some areas or the lack of healthcare facilities in others, PRESEPI was quickly overwhelmed and unable to collect samples. To address this issue, MSPP established additional specimen collection facilities, such as clinics, where nasopharyngeal swabs and saliva samples were collected from symptomatic cases and individuals planning to travel. These specimens were collected based on specific criteria, such as suspect cases matching COVID-19 case definitions and contacts of suspected and confirmed cases. To ensure effective management and accurate diagnosis of COVID-19 cases, healthcare workers were trained in the proper collection and transportation of specimens to the laboratory. These strategies facilitated COVID-19 surveillance in the region and helped to control the spread of the virus.

Positive PCR samples collected through the surveillance system were selected for sequencing based on specific criteria. To ensure adequate representation of all geographic departments, at least five samples per department were selected. Additionally, the first two confirmed cases in Haiti, both of which were imported cases, were also included in the sequencing efforts. The first two COVID-19 deaths in the country were also selected for sequencing, along with severe cases and cases with known comorbidities. These criteria would allow for a comprehensive understanding of the genetic diversity and transmission patterns of the virus in the region, providing valuable insights into its evolution and spread.

### Ethics approval

As the investigation primarily focused on routine surveillance activities and did not involve any additional procedures or interventions beyond standard diagnostic protocols, ethical approval was not required for this study.

### Viral extraction

Viral RNA was extracted from saliva and nasopharyngeal swabs using the Qiagen Qiasymphony SP DSP virus pathogen mini kit (Cat. No. 937036) with the Complex 200 offboard lysis V4 DSP protocol according to manufacturer instructions.

### Genomic sequencing

Amplification of viral genomes, library construction, and genomic analysis was done according to the protocols available at https://github.com/GhedinSGS/SARS-CoV-2_analysis. Libraries were sequenced on the Illumina NextSeq500 and NextSeq2000 using the 2 × 150 bp paired end protocol. Adapters and primers were trimmed, reads were aligned to the Wuhan/Hu-1 strain (NC_045512.2), and the 2 libraries for each sample were merged. Consensus sequences and variants were identified using the timo variant calling pipeline [10].

### Data analysis

Consensus sequences with coverage over at least 70% of the genome (20,930 nt) were classified using Pango [5] version 4.1.3, and sequences with coverage over at least 25,000 nt of the genome were included in phylogenetic analyses.

### Phylogenetic analysis

Maximum likelihood phylogenetic trees were built using the default ncov workflow (v12) in Nextstrain v4.1.1 [11], which uses Nextalign for alignment, IQ-TREE [12] 2.2.0.3 (COVID Edition) for tree building and TreeTime [13] 0.8.6 for time tree construction.

For the main phylogenetic analysis, the newly sequenced Haiti samples were placed in the context of sequences from all major global regions, focusing on North and South America because of their proximity to the Caribbean, that were publicly available in the sequence repository GISAID as of August 2022. For Caribbean countries, all sequences that were available from January-June 2020 were included. From the remaining countries, we randomly sampled 2000 sequences each from North America, South America, and the combination of Europe, Asia, Africa, and Oceania (S4 Table). Some sequences were excluded from the phylogenetic tree according to the quality control criteria of the ncov workflow. S2 Table summarizes the number of samples included per country after sample exclusion by nextstrain’s quality control. Data were visualized in R v4.1.2 using the packages ggtree [14] and tidyverse [15].

In a separate analysis where we focused on the lineage B.1.478, we collected all sequences classified as this lineage in GISAID (n = 123), which spanned dates up to April 2021 (S5 Table). To infer the probable origins of the B.1.478 lineage, an ancestral state reconstruction was performed using the Markov chain Monte Carlo (MCMC) methods available in the Bayesian Evolutionary Analysis Sampling Trees (BEAST) v.1.10.4 package [16]. A relaxed uncorrelated lognormal (UCLN) clock was used, with a Hasegawa–Kishino–Yano (HKY) model of nucleotide substitution with gamma-distributed rate variation among sites. An exponential population growth model was used during this early stage of SARS-CoV-2 epidemic growth in an immunologically naïve population. The MCMC chain was run separately four times using the BEAGLE 3 [17] library to improve computational performance, until all parameters reached convergence, as assessed visually using Tracer v.1.7.2. At least 10% of the chain was removed as burn-in, and runs for the same dataset were combined using LogCombiner v1.10.4. A MCC tree was summarized using TreeAnnotator v.1.10.4 and visualized using FigTree v.1.4.4.

### Travel data

To investigate the international spread of SARS-CoV-2, we obtained international flight passenger data from OAG (https://www.oag.com). OAG collects passenger bookings data, which accounts for true origin and destination of flight. We obtained the monthly number of passengers traveling into Haiti by air from all countries. The air passenger data used in this study are proprietary and were purchased from OAG Aviation Worldwide Ltd.

Data on international border closures was obtained from Our World in Data International travel controls dataset (https://ourworldindata.org/covid-international-domestic-travel#international-travel-controls) [8, 9].

### Maps

World map outline was generated from ggplot2 3.4.0.

## Supporting information

S1 Fig Distribution of Caribbean SARS-CoV-2 sequences against a maximum likelihood of global SARS-CoV-2 samples. (A) Maximum likelihood phylogenetic tree of samples for this study on a background of samples collected globally and publicly available on GISAID (Details on sample selection in Methods). Color represents next strain clade. (B) Bar graph of number of samples of each Nextstrain clade from each global region from the dataset used in (A), colored by region of sample collection.
(PDF)

S1 Table Pango lineage classifications and GISAID accession numbers for samples with coverage over at least 70% of the genome. These include 5 samples from October-December 2020, which were sequenced, classified, and deposited in GISAID but not included in phylogenetic analyses.
(XLSX)

S2 Table Number of GISAID sequences from each country in the phylogenetic tree in S1 Fig. After samples were removed by the ncov pipeline for quality control. Countries not listed have no samples included in the analysis.
(XLSX)

S3 Table The average number of air travels, by flight origin country, for the top 15 countries that flew into Haiti before the COVID-19 pandemic began (January–February 2020) and soon after the pandemic began (April–May 2020). Data is from OAG and is described in more details in the Methods section.
(XLSX)

S4 Table Acknowledgements table for all GISAID samples included in the phylogenetic tree in S1 Fig. Data was downloaded from GISAID on 12/22/2022. Sampling method is described in Methods section.
(XLSX)

S5 Table Acknowledgements table for all GISAID samples included in the phylogenetic tree in Fig 5. Data was downloaded from GISAID on 17/10/2022.
(XLSX)

S1 File Interactive Auspice files for viewing S1 Fig. Can view the phylogenetic tree and annotate based on metadata using Auspice (https://auspice.us/) by dragging all **S1 File** onto the webpage.
(ZIP)

S2 File Interactive Taxonium files for viewing S1 Fig. Can view the phylogenetic tree and annotate based on metadata using Taxonium (https://auspice.us/) by dragging both **S2 File** onto the webpage then clicking “launch taxonium”.
(ZIP)

S1 Checklist Inclusivity in global research. (DOCX)
