# Supplementary figures and images for: Genomic analysis of the early COVID-19 pandemic in Haiti reveals Caribbean-specific variant dynamics
Source: PLOS Glob Public Health. 2024 Nov 20;4(11):e0003536. doi: 10.1371/journal.pgph.0003536 (PMC11578445; doi:10.1371/journal.pgph.0003536)

**A**

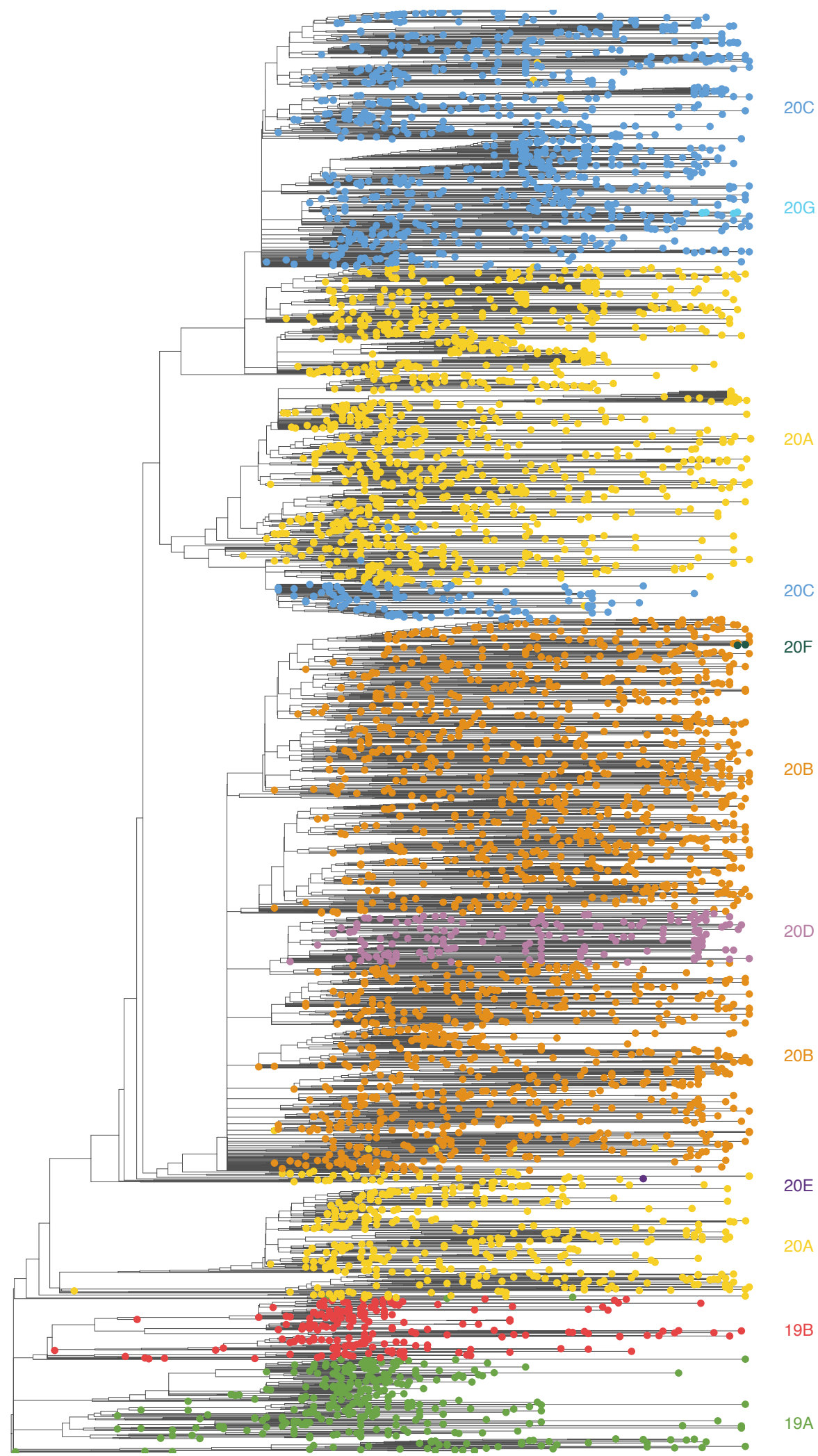

## B

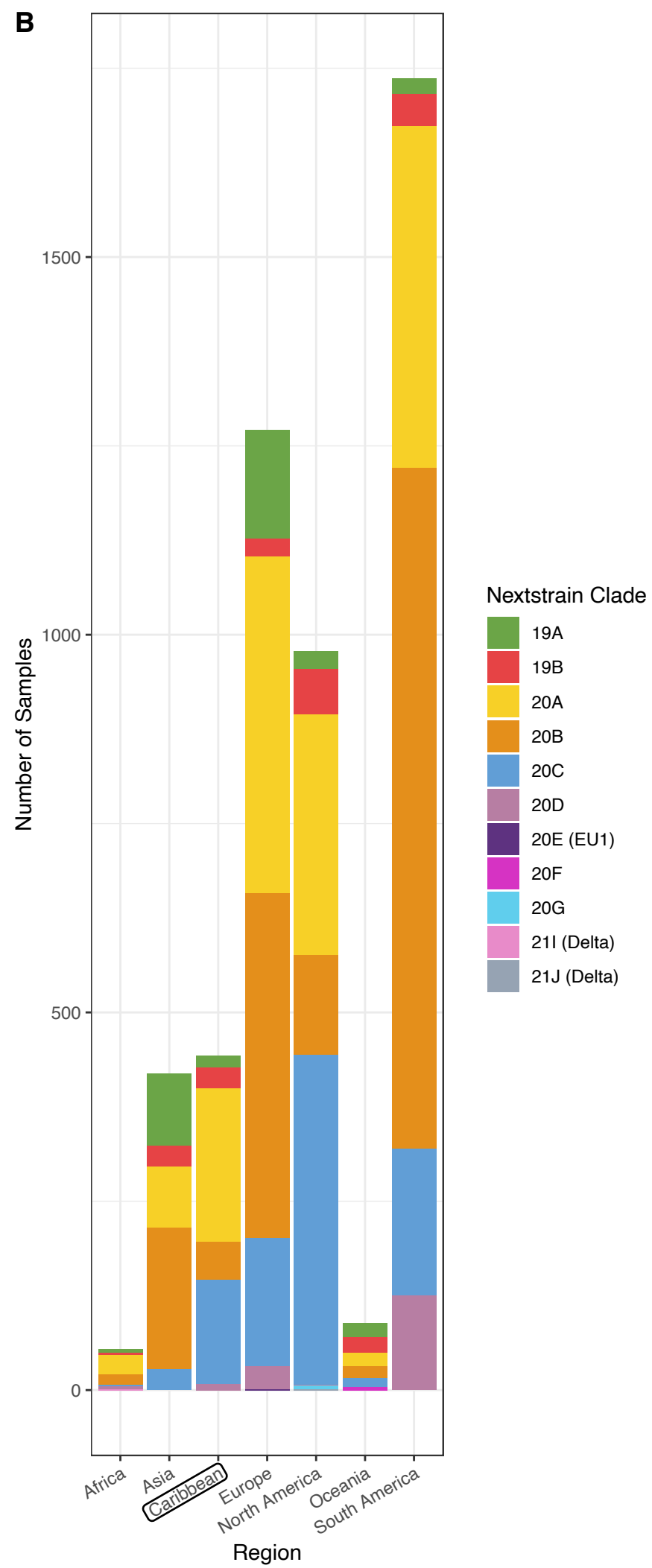

Supplement: S1 Fig — (A) Maximum likelihood phylogenetic tree of samples for this study on a background of samples collected globally and publicly available on GISAID (Details on sample selection in Methods). Color represents next strain clade. (B) Bar graph of number of samples of each Nextstrain clade from each global region from the dataset used in (A), colored by region of sample collection. (PDF) [file pgph.0003536.s001.pdf]
